# Effect of Varying Sintering Speed on Optical Characteristics Alteration of Different Yttria-Doped Monochromatic Partially Stabilized Zirconia: An *In Vitro* Study

**DOI:** 10.1055/s-0045-1809181

**Published:** 2025-05-21

**Authors:** Atthasit Boonbanyen, Niwut Juntavee, Apa Juntavee

**Affiliations:** 1 Division of Biomaterials and Prosthodontics Research, Faculty of Dentistry, Khon Kaen University, Khon Kaen, Thailand; 2 Department of Prosthodontics, Faculty of Dentistry, Khon Kaen University, Khon Kaen, Thailand; 3 Division of Pediatric Dentistry, Department of Preventive Dentistry, Faculty of Dentistry, Khon Kaen University, Khon Kaen, Thailand

**Keywords:** color appearance, contrast, sintering rate, translucency, opalescence, zirconia

## Abstract

**Objectives:**

Sintering influences the color of zirconia. This study assessed the influence of varying sintering rates on optical characteristics of 3, 4, and 5 mol% yttria (Y) containing monochromatic zirconia.

**Materials and Methods:**

A total of 135 bar specimens (width × length × thickness = 11.2 × 20 × 1.5 mm) were prepared from monochromatic 3Y, 4Y, and 5Y zirconia, and randomly sintered at regular (RS: 10°C/min), fast (FS: 35°C/min), and speed (SS: 70°C/min) sintering rate ( *n* = 15/group). Translucency parameter (TP _00_ ), contrast ratio (CR), opalescence parameter (OP), and color difference (∆E _00_ ) were evaluated with the CIEL*a*b* system. Microstructure, crystalline [monoclinic (m), tetragonal (t), and cubic (c)] phases, and surface roughness (Ra) were evaluated by scanning electron microscope (SEM), X-ray diffraction (XRD), and three-dimensional digital microscopy.

**Statistical Analysis:**

Analysis of variance and Bonferroni comparisons were determined for significant differences ( *α* = 0.05).

**Results:**

Significant differences in TP _00_ , CR, OP, and ∆E _00_ upon zirconia types and sintering rates were indicated ( *p* < 0.05). Significant increasing TP _00_ , whereas decreasing CR and OP was shown upon increasing the Y content (5Y > 4Y > 3Y), and speeding sintering (SS ≅ FS > RS). Significant increasing ∆E _00_ upon increasing the Y content (5Y > 4Y > 3Y) was shown but was within an acceptable threshold (∆E _00_ ≤ 1.8). Ra was higher for 3Y > 4Y > 5Y. SEM indicated a larger grain for 5Y > 4Y > 3Y. XRD indicated higher *t* -phase in 3Y, whereas higher *c* -phase in 5Y.

**Conclusion:**

Increasing translucency, whereas reducing contrast and opalescence were affected by the amount of Y content (5Y > 4Y > 3Y) and speeding sintering rate (SS ≅ FS > RS). Increasing color alteration was affected by the amount of Y content (5Y > 4Y > 3Y), but was within acceptable limits, suggesting rapid sintering rate to achieve better optical characteristics.

## Introduction

The esthetic requirement of dental restorations has been navigating the improvement of numerous new ceramic materials to provide restorations with a lifelike appearance. The glass-based ceramics were originally introduced for reestablishing the severely damaged teeth in the esthetic zone on account of their high translucence appearances. Contrariwise, the low toughness and strength properties of glass ceramics have limited their use only for the restoration from partial coverage to full coverage restorations, and short-span bridges for the anterior teeth. The zirconia dental ceramics were contemporarily developed to overcome the less strength of the glass ceramics, which are feasibly utilized for the fabrication of long-span restoration, especially in a high-loading region. 1 Different types of novel zirconia were developed based on the difference in crystalline structures. Pure zirconia typically appears in three distinctive crystalline phases relating to the surrounding temperatures. The monoclinic ( *m* ) phase is the only form that remains constant at room temperature till reaching 1,170°C, the tetragonal ( *t* ) phase is established, and eventually converts to the cubic ( *c* ) phase as the temperature rises to 2,370°C. Quite the opposite, the *c* -phase is consecutively reversed to the *t* -phase and the *m* -phase as the zirconia cools down to room temperature. To formulate the *t* - and *c* -phase at room temperature, the yttrium oxide (Y _2_ O _3_ ) dopant was generally included, ranging from 3 to 5 mol% yttria (Y)-doped zirconia, through the manufacturing process. The 3 mol% yttria-stabilized tetragonal zirconia polycrystal (3Y-TZP) was primarily developed that predominantly consists of the *t* -phase, which possessed an extreme strength from the phase transformation ( *t* → *m* phase) mechanism, resulting in an approximately 4 to 5% volumetric expansion of the zirconia grain, enabled generating the compressive stress to inhibit crack propagation. 2 Still, its dull-white optical appearance and high opacity are the disadvantages of possessing a small grain size, large grain boundaries, and high quantities of aluminum oxides. Therefore, it is commonly used as a substructure for veneering with glass ceramics to imitate the natural tooth appearance. Yet, the delamination of veneering ceramic from the zirconia substrate is a major detriment. Subsequently, the monolithic 3Y-TZP was introduced with excellent fracture resistance and improved translucence through the smaller refining grain size than the archetypes together with reducing the quantities of alumina, which could reduce the scattering effect from the alumina contents, mostly located at the zirconia grain boundaries. Nevertheless, it still possesses unsuitable translucence for restorations in the esthetic zone. 3 The extreme translucency zirconia is contemporarily industrialized by adding 4 to 5 mol% of Y _2_ O _3_ to produce the 4 to 5 mol% partially stabilized zirconia (4Y-PSZ and 5Y-PSZ), which comprise more *c* -phases based on the amount of Y stabilizer. Both 4Y-PSZ and 5Y-PSZ afford better translucency than 3Y-TZP because the isotropic structure of the *c* -phase dominates the light refraction to be in a straighter line than the asymmetrical structure of the *t* -phase. Likewise, they offer a larger grain size with lower grain boundaries, which induces less light scattering. The alteration of the sintering process, for instance, the increasing sintering temperature and prolonged sintering time have been reported to be capable of enhancing translucency of the different types of monolithic zirconia, which dramatically benefits clinicians in the fabrication of dental restorations. 4 5 6

In achieving esthetics restoration, the selection of ceramic for fabrication is a crucial concern, that needs to consider the optical properties of the material, including translucency, contrast, opalescence, and color perception. 7 8 9 The translucency is defined as the amount of light transmission through the material and was reflected in terms of the translucency parameter (TP _00_ ) and contrast ratio (CR). 10 An extremely translucent material would exhibit a superior TP _00_ value, but a lesser CR value, since both parameters are contrary correlations. 11 The translucency of zirconia is associated with the microstructure including the grain size, the composition and arrangement of chemical elements, the relative phase distribution, and the external surface topography. 12 13 14 15 The low translucency zirconia is better for masking the color of the underlying substrate. 16 17 The optical phenomenon of the visible light path scattering and transmitting on the zirconia is related to the dimensions of grains, crystalline microstructures, coloring pigments, and internal porosities of material. 18 The longer wavelengths (orange to red) can travel through the materials, while the shorter wavelengths (purple to blue) seem to scatter on the surface. The restoration would look bluish once the light is redirected from it and appear orange hue as the light transmits right through. This existence is recognized as opalescence and is measured in terms of the opalescence parameter (OP), which produces the restoration closely imitating the lifelike human enamel (OP ≈19.8–27.6). 10 19 The opalescence of the zirconia could be improved by incorporating some metal oxides, for instance, ZrO _2_ , Y _2_ O _3_ , SnO _2_ , and V _2_ O _5_ , and inducing the larger zirconia grain than the visible light wavelength. 20 21 Regarding color perception, the color difference (∆E _00_ ) is used to clarify the level of color alteration, which is based on the perceptibility threshold (PT, ∆E _00_ = 0.81) and the acceptability threshold (AT, ∆E _00_ = 1.80). The ∆E _00_ < 0.81 denoted “ *clinically indifferent* ,” ∆E _00_ = 0.81 to 1.80 denoted “ *clinically acceptable* ,” and ∆E _00_ > 1.80 denoted “ *clinically unacceptable* ” perception of color difference. 7 17 The more ∆E _00_ increases, the less acceptability in color alteration.

Zirconia sintering is a critical process to accomplish esthetics and durable restorations, which depends on the sintering rate, sintering temperature, sintered holding time, and cooling rate. The sintering rate is a crucial parameter to generate heat per minute (°C/min) to the zirconia microstructures until reaching the mature sintering temperature. The sintering rates were customarily accomplished between 5 and 20°C/min based on the manufacturer's instruction, which is a time-consuming and energy-consuming process. 22 Shortening the sintering time is not only advantageous for the dental technicians but also favors clinicians for efficiently rendering the chairside zirconia restoration. 5 Several efforts were commenced to enhance the superior optical characteristics of zirconia restoration through the manufacturing processes, for instance, adjusting microstructure, adding some chemical elements, and distributing relative phase content. *Vis-a-vis* , it is widespread to accomplish by adjusting the sintering procedures. 3 4 5 11 22 23 24 25 26 27 28 Principally, shortened sintering time with rapid sintering rate is an appealing method, however, the influence on optical characteristics of different types of zirconia remains unclear due to limited studies. 9 29 The authors are unaware of a study reported on the comparison of heating rate alteration effect on the optical characteristics of the various types of the monochromatic 3Y, 4Y, and 5Y %mol yttria-doped zirconia. Hence, this study aimed to determine the influences of zirconia types, sintering rate, and their combinative effects on optical characteristics. The null hypothesis was that there was no significant difference in optical characteristics including translucency, contrast, opalescence, and color difference upon different Y-containing zirconia, sintering rate, and their combinative interactions.

## Materials and Methods

The study trailed the Checklist for Reporting In-vitro Studies standards for *in vitro* study. The appraised sample size was estimated using the G*power 3.1 software (Heinrich Heine Universität, Düsseldorf, Germany) according to the statistical data from the former study 30 at powers of test = 0.9, and *α* -error = 0.05, as shown in Eq. (1) .



where: *Z _α_*_=_ standard normal deviation = 1.96 ( *α* error = 0.05), *Z _β_*_=_ standard normal deviation = 1.28 ( *β* error = 0.1), *µ*_1_ - *µ*_2_ = mean difference between experimental group = 0.02, and *σ = s* tandard deviation (SD) ( *σ*_1_ = 0.02, *σ*_2_ = 0.01). A sample size of 15 specimens per group was used for this experiment.

## Preparation of the Zirconia Specimens

The monochromatic, the VITA classical (Vita Zahnfabrik) shade A2, presintered 3Y-TZP, 4Y-PSZ, and 5Y-PSZ zirconia blanks (Bloomden Bioceramics, Hunan, China), were sectioned into bar-shaped specimens at an enlarged dimension (width × length × thickness = 14 × 25 × 1.8 mm) to counteract for sintering shrinkage using a diamond-coated wheel in a sectioning apparatus (Mecatome T180, Presi, Eybens, France). The specimens were ground with silicon carbide abrasive paper up to grit 7,000 and subsequently polished with 1-μm diamond suspension in a polishing machine (Ecomet3, Beuhler, Lake Bluff, Illinois, United States) to achieve a smooth surface. All specimens were cleaned with distilled water to eliminate debris and dried in a desiccator (Ailite GP5, Ailite, Guangdong, China) for 24 hours. The specimens were randomly allocated into nine groups ( *n* = 15) according to the types of zirconia and sintering rate (regular [RS: 10°C/min], fast [FS: 35°C/min], speed [SS: 70°C/min]) ( Table 1 ). The sintering process was accomplished in the sintering furnace (inFire HTC, Dentsply Sirona, Bensheim, Germany) at the allocated sintering speed until reaching 1,530°C of sintering temperature with 120 minutes of holding time, and cooled down at –10°C/min of cooling rate. Once sintering was completed, the specimen was measured with a measuring device (Mitutoyo, Tokyo, Japan) to derive the final specimen dimension (width × length × thickness = 11.2 × 20 × 1.5 mm) with an accuracy of ± 1 µm.

**Table 1:** Zirconia type, brand/manufacturers, composition (wt%), batch number, of the monochromatic 3 mol% yttria-stabilized tetragonal zirconia polycrystal (3Y-TZP), 4 and 5 mol% yttria-partially stabilized zirconia (4Y-PSZ, 5Y-PSZ) sintered with regular (RS; 10°C/min), fast (FS; 35°C/min), and speed (SS; 70°C/min) heat rate, abbreviation (Abv.) of groups

Zirconia					Rate	Group Abv.
Type	Brand/Manufacturer	Composition	Batch no.	Abv.		
3 mol% yttria-stabilized tetragonal zirconia polycrystal (3Y-TZP)	Bloomden ST Mu, Bloomden Bioceramics, Hunan, China	≥ 99% ZrO _2_ + HfO _2_ + Y _2_ O _3,_ 4.5–6% Y _2_ O _3,_ < 0.5% Al _2_ O _3_ < 0.5% other oxides	S2519121921946	3Y	RS	3YRS
				3Y	FS	3YSF
				3Y	SS	3YSS
4 mol% yttria-partially stabilized zirconia (4Y-PSZ)	Bloomden ST-Plus Mu, Bloomden Bioceramics, Hunan, China	≥ 99% ZrO _2_ + HfO _2_ + Y _2_ O _3,_ 7–7.8% Y _2_ O _3,_ < 0.15% Al _2_ O _3_ < 0.15% other oxides	S2519080921916	4Y	RS	4YRS
				4Y	FS	4YSF
				4Y	SS	4YSS
5 mol% yttria-partially stabilized zirconia (5-PSZ)	Bloomden UT Mu, Bloomden Bioceramics, Hunan, China	≥ 99% ZrO _2_ + HfO _2_ + Y _2_ O _3,_ 9–10% Y _2_ O _3,_ < 0.05% Al _2_ O _3_ < 0.05% other oxides	S2619072241942	5Y	RS	5YRS
				5Y	FS	5YFS
				5Y	SS	5YSS

## Determination of the Optical Characteristics

The optical characteristics of monochromatic zirconia specimens with different sintering rates were achieved using a spectrophotometer (ColorQuest XE, Hunter, Reston, Virginia, United States) by setting the parameters at 10-degree observer angle, 100% ultraviolet, D-65 illuminant at the standard wavelength between 380 and 780 nm, and 4 mm diameter of the aperture. The device was calibrated with a standard white tile before starting the measurements. A transparent acrylic template was employed to maintain the position of the specimen during the optical parameter measurements, which were determined independently at the mid portion of the left, central, and right sides for each specimen. The Commission International de I'Eclairage (CIE L*a*b*) color space system was used to determine for L*, a*, and b* color parameters, which were attained for the lightness, the red-green coordinate, and the yellow-blue coordinate of the specimens, respectively, against the white (W) (L* _W_ = 96.70, a* _W_ = 0.10, b* _W_ = 0.20) and black (B) (L* _W_ = 30.53, a* _W_ = 0.95, b* _W_ = 0.36) background. The CIEDE2000 was used to determine for TP _00_ , CR, OP, and color difference (∆E _00_ ). The relative TP _00_ values were calculated from the differences between color determinants on black and white backgrounds, using Eq. (2) .



where: the *L* ′, *C* ′, and *H* ′ represent the differences in lightness, chroma, and hue of the specimens against black (B) and white (W) background; *R _T_* is the rotational function that accounts for the interaction between chroma and hue difference in blue region; *S _L_* , *S _C_* , and *S _H_* are the weighting functions for lightness, chroma, and hue; and *K _L_* , *K _C_* , and *K _H_* are the correct term for experimental conditions, which were set at 1 in the present study.

The CR values were determined from Eqs. (3) and (4) , which ranged from 0.0 (transparent) to 1.0 (perfectly opaque). In the tristimulus color space, *Y* represents the brightness illuminance; *Y _B_* and *Y _w_* are the values of a specimen placed on the black and white backgrounds, respectively; and *Y _n_* is equal to 100.





The OP values were determined by using Eq. (5) .



The ∆E _00_ values were calculated from the data set of each specimen on a standard white background, compared with the mean coordinate of the same type of zirconia that was sintered at RS sintering rate, as Eq. (6) .



where the *L* ′, *C* ′, and *H* ′ represent the differences in the lightness, chroma, and hue of a set of samples.

## Determination of the Microstructure and Chemical Composition

Three specimens represented as-sintered surfaces were randomly selected from each group for microscopic examination. The specimens were cleaned with distilled water, dried in the auto-desiccator at normal ambient temperature for 24 hours, and then coated with gold-palladium in a sputter coater (K500X, Quorum Technology, Kent, United Kingdom) at 10 mA current, 130 m-Torr vacuum, for 3 minutes. The specimen surfaces were examined for grain morphology and grain size with a scanning electron microscope (SEM, SU3800, Hitachi, Tokyo, Japan) and grain size analyzer program (GSA program, KKU, Khon Kaen, Thailand) at ×10K magnification. The chemical compositions were characterized with energy dispersive spectroscopy (EDS, Oxford, High Wycombe, United Kingdom).

## Determination of the Phase Composition

The fraction of *c* -, *t* -, and *m* -phases of all specimen groups was observed using X-ray diffraction (XRD, Bruker, Karlsruhe, Germany). The zirconia specimens' surface was inspected using Cu k-α radiation at a diffraction angle (2θ) of 20 to 90 degrees, with a step size of 0.02 degrees for a second interval. The XRD patterns were generated using Origin-Pro 2019 (OriginLab, Wellesley, Massachusetts, United States) to analyze the relative proportions of phases based on the peak intensity using the Match-3.0 software (Crystal Impact, Bonn, Germany). The peaks were cross-referenced to the Joint Committee of Powder Diffraction Standards database files (PDFs) No. 03–0640, 02–0733, and 07–0343, for *c* -, *t* -, and *m* -phase, respectively. The relative intensities of peaks for *m* -phase ( *I _m_* ), *t* -phase ( *I _t_* ), and *c* -phase ( *I _c_* ) were analyzed by the X'Pert–Plus software (Philips, Almelo, Netherlands). The calculation was performed upon matching a Pseudo-Voigt distribution to the courtesy peak and considering the area beneath the curve. Considering the influence of yttria on the lattice parameters, the corrected factor of 1.311 was used to calibrate the nonlinear curve of assimilated intensity ratios against volume fraction. The Garvie–Nicholson formula was applied for calculating the proportion of *m* -phase ( *X _m_* ), *t* -phase ( *X _t_* ), and *c* -phase ( *X _c_* ) as Eqs. (7) , (8) , and (9) .







## Determination of the Surface Topography

The surface topography and surface roughness of the zirconia specimens were examined with the three-dimensional (3D) digital microscope (Olympus DSX1000, Evident, Tokyo, Japan), and further analyzed with the image analysis software (PRECiV-Olympus, Evident). The bright-field mode at ×79 magnification with high contrast and high dynamic range texture was selected to evaluate the 3D topography at the area 3.6 × 3.6 mm ^2^ , for five areas of specimen in each group, and calculated for the average surface roughness (Ra).

## Statistical Analysis

The data were executed with the Shapiro–Wilk test for normality test, and Levene's test for homoscedasticity test using statistical software (IBM SPSS V-26, SPSS, Chicago, Illinois, United States). Since the data were normally distributed and presented homoscedasticity ( *p* > 0.05), the two-way analysis of variance (ANOVA) and *post hoc* Bonferroni multiple comparisons were performed to detect substantial variations in optical characteristics (TP _00_ , CR, OP, and ∆E _00_ ) of the 3, 4, and 5 mol% yttria containing monochromatic zirconia, upon different sintering rate. A statistically significant difference was set at *p* < 0.05. Descriptive analysis was employed to assess the grain size, elemental composition, relative phases composition, and surface roughness of the zirconia.

## Results

**Fig. 1:**
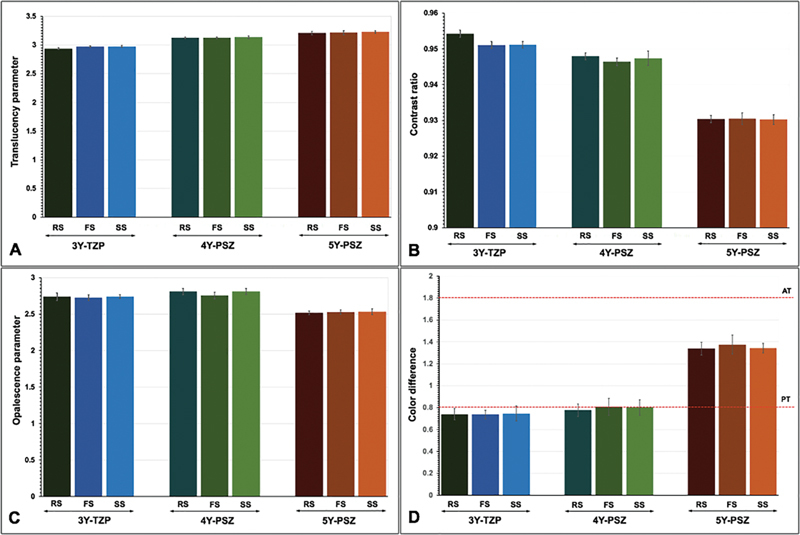
Translucency parameter ( **A** ), contrast ratio ( **B** ), opalescence parameter ( **C** ), and color difference ( **D** ) within the perceptible threshold (PT) and acceptable threshold (AT) of monochromatic 3Y-TZP, 4Y-PSZ, and 5Y-PSZ upon sintered at regular (RS), fast (FS), and speed (SS) sintering rate.

**Fig. 2:**
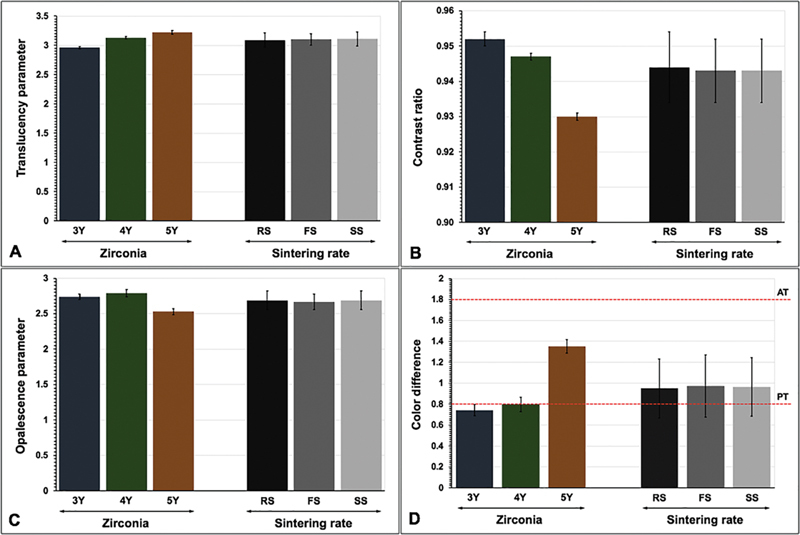
Influence of type of zirconia (3Y-TZP, 4Y-PSZ, and 5Y-PSZ) and sintering rate (regular [RS], fast [FS], and speed [SS]) on translucency parameter ( **A** ), contrast ratio ( **B** ), opalescence parameter ( **C** ), and color difference ( **D** ) within perceptible (PT) and acceptable threshold (AT).

The mean and SD of TP _00_ , CR, OP, ∆E _00_ , Ra, grain size distribution, relative phase content, and chemical elements of 3Y, 4Y, and 5Y-contained monochromatic fully stabilized zirconia upon RS, FS, and SS sintering rate were reported ( Table 2 and Figs. 1 2 3 ). Two-way ANOVA indicated that the color parameters, including TP _00_ , CR, OP, and ΔE _00_ , were significantly influenced by zirconia type, sintering rate, and the interaction of zirconia type and sintering rate ( *p* < 0.05), except ΔE _00_ for the factor of sintering rate, and the interaction of two factors ( *p* > 0.05) ( Table 3 ). *Post hoc* Bonferroni multiple comparisons indicated that types of zirconia and sintering rate presented a statistically significant effect on the TP _00_ , CR, OP, and ΔE _00_ ( *p* < 0.05), except for groups of RS/FS in TP _00_ and CR, RS/FS, and RS/SS in OP, and RS/FS/SS in ΔE _00_ ( *p* > 0.05) ( Table 4 and Fig. 2 ). Considering the type of zirconia, the study suggested that raising the amount of yttria-containing in zirconia significantly increased TP _00_ , and ΔE _00_ , whereas significantly decreased CR and OP ( *p* < 0.05) ( Table 4 and Fig. 2 ). Regarding sintering rate, the study indicated that sintering zirconia with either FS or SS resulted in significant increase in TP _00_ , but significant reduction in CR compared with RS ( *p* < 0.05). Yet, no significant difference in TP _00_ and CR between FS/SS, no significant difference in OP between RS/FS and RS/SS, and no significant difference in ΔE _00_ between RS/FS/SS ( *p* > 0.05) were observed ( Table 4 and Fig. 2 ). Concerning the color alteration, the study indicated that 5Y revealed a significantly higher color alteration than 4Y and 3Y, respectively, suggesting that increasing Y in zirconia exhibited a significantly easier color alteration ( *p* < 0.05) ( Table 4 and Fig. 2D ). The study indicated that the sintering at either RS, FS, or SS sintering rate produced no significant effect on color alteration ( *p* > 0.05) ( Table 4 and Fig. 2D ). Nevertheless, color alteration for all groups was between PT (ΔE _00_ ≤ 0.8) and AT (ΔE _00_ ≤ 1.8) ( Fig. 1D ). Color alteration of zirconia upon different types of zirconia and different sintering rates was within the AT ( Fig. 2D ).

**Table 2:** Mean, standard deviation (SD) of translucency parameter (TP _00_ ), contrast ratio (CR), opalescence parameter (OP), color difference (∆E _00_ ), surface roughness (Ra), percentage of fine (f), medium (m), and large (l) grain size distribution (%), relative cubic ( *c* -), tetragonal ( *t* -), and monoclinic ( *m* -) phase content (wt.%), and chemical elements (wt.%) of monochromatic 3Y-TZP, 4Y-PSZ, and 5Y-PSZ upon sintered at regular (RS), fast (FS), and speed (SS) sintering rate

Group		*n*	TP _00_	CR	OP	**∆** E _00_	Ra	Grain size	Phase	Chemical element
Type	Rate		Mean ± SD	Mean ± SD	Mean ± SD	Mean ± SD	Mean ± SD	*f* / *m* / *l*	*c* - / *t* - / *m* -	Zr / O / Y / Al Os / Hf / Th / Fe
3Y	RS	15	2.94 ± 0.02	0.95 ± 0.01	2.74 ± 0.05	0.74 ± 0.05	5.72 ± 1.16	93.9 / 6.1 / 0.0	20.6 / 76.7 / 2.7	74.3 / 18.1 / 0.8 / 0.1 3.6 / 1.8 / 1.2 / 0.08
3Y	FS	15	2.97 ± 0.01	0.95 ± 0.01	2.73 ± 0.04	0.74 ± 0.04	4.67 ± 1.19	94.3 / 5.7 / 0.0	16.1 / 78.2 / 5.7	74.5 / 17.9 / 0.6 / 0.1 3.9 / 1.9 / 1.1 / 0.07
3Y	SS	15	2.98 ± 0.02	0.95 ± 0.01	2.74 ± 0.03	0.75 ± 0.07	3.98 ± 0.53	93.5 / 6.5 / 0.0	19.2 / 77.3 / 3.5	75.1 / 17.1 / 0.6 / 0.1 3.8 / 1.9 / 1.4 / 0.08
4Y	RS	15	3.13 ± 0.01	0.95 ± 0.01	2.81 ± 0.04	0.78 ± 0.06	4.14 ± 0.74	72.6 / 26.6 / 0.8	35.5 / 63.8 / 0.7	73.8 / 17.0 / 2.1 / 0.1 3.7 / 1.8 / 1.4 / 0.08
4Y	FS	15	3.12 ± 0.02	0.95 ± 0.01	2.76 ± 0.05	0.81 ± 0.08	4.03 ± 1.09	75.2 / 24.8 / 0.0	36.4 / 63.3 / 0.3	73.7 / 16.7 / 2.3 / 0.1 3.7 / 1.8 / 1.6 / 0.09
4Y	SS	15	3.14 ± 0.02	0.95 ± 0.02	2.81 ± 0.04	0.80 ± 0.07	4.13 ± 0.82	72.8 / 27.2 / 0.0	37.3 /62.5 / 0.2	73.4 / 17.1 / 2.3 / 0.1 3.7 / 1.9 / 1.4 / 0.09
5Y	RS	15	3.21 ± 0.03	0.93 ± 0.01	2.52 ± 0.03	1.34 ± 0.06	3.58 ± 1.09	26.9 / 53.9 / 19.2	55.0 / 44.6 / 0.4	71.4 / 16.1 / 4.4 / 0.1 3.7 / 1.8 / 2.4 / 0.08
5Y	FS	15	3.21 ± 0.04	0.93 ± 0.01	2.53 ± 0.03	1.37 ± 0.09	3.30 ± 1.73	29.8 / 45.5 / 24.7	58.0 /41.1/ 0.9	71.5 / 15.7 / 4.7 / 0.1 3.3 / 1.9 / 2.8 / 0.08
5Y	SS	15	3.23 ± 0.02	0.93 ± 0.01	2.53 ± 0.04	1.34 ± 0.04	3.30 ± 0.87	30.3 / 48.7 / 21.1	54.4 / 45.3 / 0.3	72.0 / 15.6 / 4.5 / 0.1 3.3 / 1.9 / 2.5 / 0.07

**Fig. 3:**
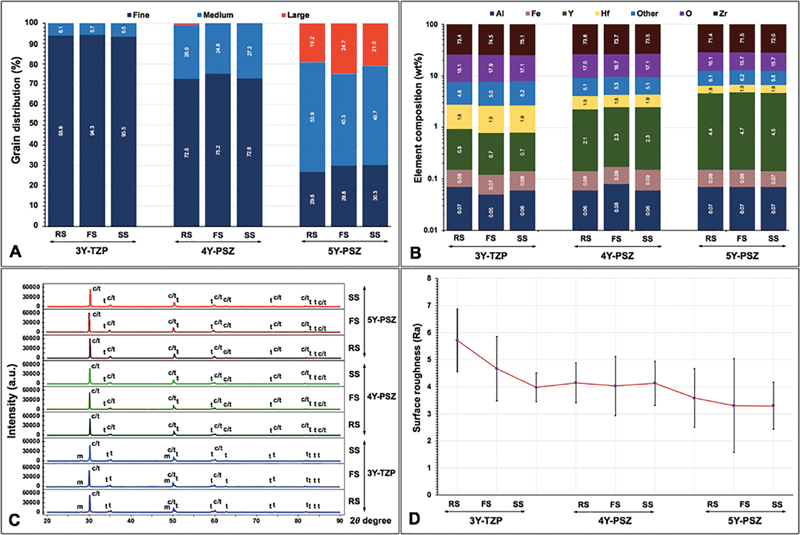
( **A** ) Grain distribution, ( **B** ) elemental composition, ( **C** ) X-ray diffraction pattern, and ( **D** ) surface roughness of monochromatic 3Y-TZP, 4Y-PSZ, and 5Y-PSZ upon sintered at regular (RS), fast (FS), and speed (SS) sintering rate.

**Table 3:** Two-way ANOVA of (a) translucency parameter (TP _00_ ), (b) contrast ratio (CR), (c) opalescence parameter (OP), (d) color difference (∆E _00_ ) of monochromatic 3Y-TZP, 4Y-PSZ, and 5Y-PSZ upon sintered at regular (RS), fast (FS), and speed (SS) sintering rate

(a) ANOVA of TP _00_ upon different factors					
Source	SS	df	MS	*F*	*p*
Corrected model	1.523	8	0.190	398.196	< 0.001
Intercept	1299.397	1	1299.397	2718463.543	< 0.001
Zirconia types	1.504	2	0.752	1572.936	< 0.001
Sintering rates	0.011	2	0.006	11.944	< 0.001
Zirconia types * Sintering rates	0.008	4	0.002	3.952	0.005
Error	0.060	126	0.000		
Total	1300.980	135			
Corrected total	1.583	134			
**(b) ANOVA of CR upon different factors**					
Corrected model	0.012	8	0.001	826.619	< 0.001
Intercept	120.101	1	120.101	66998730.9	< 0.001
Zirconia type	0.012	2	0.006	3273.240	< 0.001
Sintering rate	0.0000649	2	0.00003245	18.103	< 0.001
Zirconia type * Sintering rate	0.00005425	4	0.00001356	7.566	< 0.001
Error	0.001	126	0.000001793		
Total	120.114	135			
Corrected total	0.012	134			
**(c) ANOVA of OP upon different factors**					
Corrected model	1.814	8	0.227	139.857	< 0.001
Intercept	973.326	1	973.326	600191.191	< 0.001
Zirconia types	1.780	2	0.890	548.739	< 0.001
Sintering rates	0.014	2	0.007	4.347	0.015
Zirconia types * Sintering rates	0.021	4	0.005	3.171	0.016
Error	0.204	126	0.002		
Total	975.345	135			
Corrected total	2.019	134			
**(d) ANOVA** ∆ ** E _00_ upon different factors **					
Corrected model	10.301	8	1.288	318.158	< 0.001
Intercept	125.185	1	125.185	30930.530	< 0.001
Zirconia types	10.281	2	5.141	1270.128	< 0.001
Sintering rates	0.011	2	0.006	1.406	0.249
Zirconia types * Sintering rates	0.009	4	0.002	0.549	0.700
Error	0.51	126	0.004		
Total	135.997	135			
Corrected total	10.811	134			

Abbreviations: ANOVA, analysis of variance; df, degree of freedom; *F* , *F* -ratio; MS, mean square; SS, sum of squares.

**Table 4:** *Post hoc* Bonferroni multiple comparisons of (a) translucency parameter (TP _00_ ), (b) contrast ratio (CR), (c) opalescence parameter (OP), (d) color difference (∆E _00_ ) of monochromatic 3Y-TZP, 4Y-PSZ, and 5Y-PSZ upon sintered at regular (RS), fast (FS), and speed (SS) sintering rate

**(a)** ***Post hoc*** ** multiple comparison of TP _00_ as a function of zirconia and sintering rate **							
**Zirconia**	**3Y**	**4Y**	**5Y**	**Sintering rate**	**RS**	**FS**	**SS**
3Y	1	0.001	0.001	RS	1	0.018	0.001
4Y		1	0.001	FS		1	0.121
5Y			1	SS			1
**(b)** ***Post hoc*** **multiple comparison of CR as a function of zirconia and sintering rate**							
**Zirconia**	**3Y**	**4Y**	**5Y**	**Sintering rate**	**RS**	**FS**	**SS**
3Y	1	0.001	0.001	RS	1	0.001	0.001
4Y		1	0.001	FS		1	1
5Y			1	SS			1
**(c)** ***Post hoc*** **multiple comparison of OP as a function of zirconia and sintering rate**							
**Zirconia**	**3Y**	**4Y**	**5Y**	**Sintering rate**	**RS**	**FS**	**SS**
3Y	1	0.001	0.001	RS	1	0.078	1
4Y		1	0.001	FS		1	0.019
5Y			1	SS			1
**(d)*Post hoc*multiple comparison of** ∆ ** E _00_ as a function of zirconia and sintering rate **							
**Zirconia**	**3Y**	**4Y**	**5Y**	**Sintering rate**	**RS**	**FS**	**SS**
3Y	1	0.001	0.001	RS	1	0.290	1
4Y		1	0.001	FS		1	1
5Y			1	SS			1

**Fig. 4:**
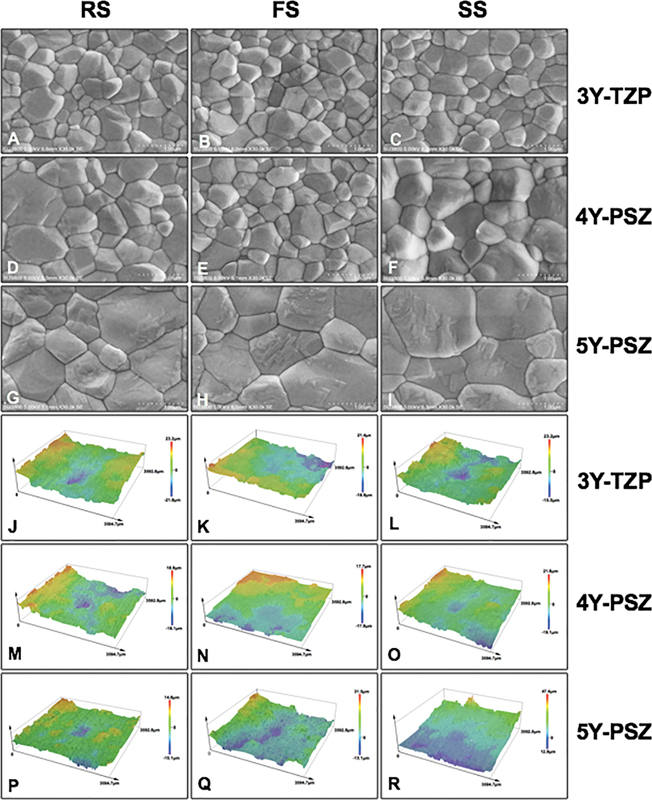
Scanning electron microscope photomicrographs at ×30K magnification indicated grain size and grain distribution ( **A** – **I** ), and surface topography indicated surface roughness ( **J** – **R** ) of monochromatic 3Y-TZP [( **A** – **C** ); ( **J** – **L** )], 4Y-PSZ [( **D** – **F** ); ( **M** – **O** )], and 5Y-PSZ [( **G** – **I** ); ( **P** – **R** )] upon sintered at regular [RS; ( **A** , **D** , **G** , **J** , **M** , and **P** )], fast [FS; ( **B** , **E** , **H** , **K** , **N** , and **Q** )], and speed [SS; ( **C** , **F** , **I** , **L** , **O** , and **R** )] sintering rate.

The SEM photomicrographs at ×10K magnification were quantified for percentages (%) grain size distribution as fine grains (F, 0.01–0.99 µm), medium grains (M, 1.00–1.99 µm), and large grains (L, 2.00–2.99 µm) ( Table 2 and Fig. 3A ). All 3Y groups demonstrated mainly F grain and a small amount of M grain. All 4Y groups principally comprised of F grain and a minor amount of M grain. All 5Y groups were composed of M grains more than F and L grains. However, respectively higher percentages of M grain in 5Y than 4Y and 3Y groups were indicated. Both 3Y and 4Y groups were rarely composed of the L grain. The grain size distribution in the same type of zirconia was not influenced by the sintering rate. Notably, 3Y demonstrated the densely packed F grain with a mostly round shape appearance, whereas 4Y presented the mixing of the F grain with a round shape circumferentially located around M grain with polygonal shape appearance, while 5Y mostly presented with dense compaction L grain with polygonal shape appearance ( Fig. 4A–I ). The chemical element composition (wt.%) for all groups of zirconia comprised zirconia (Zr) and oxygen (O) as principal elements. Besides, yttria (Y), aluminum (Al), osmium (OS), hafnium (Hf), thorium (Th), and ferrous (Fe) were minor elements. The Y element was diversified according to the type of zirconia. The 5Y groups exhibited the highest Y (4.4–4.7%), whereas the 3Y groups exhibited the lowest Y (0.7–0.8%). Varying sintering rates did not significantly alter the percentage of chemical elements for all zirconia types ( Table 2 and Fig. 3B ).

The XRD pattern for all zirconia groups was composed of *c* -, *t* -, and *m* -phases ( Table 2 and Fig. 3C ). The main peak of the *c* -phase was located at the diffraction angle (2θ) of 30.168 degrees, and belonged to the *111-* crystalline plane, while the minor peaks were found at 2θ of 35.023, 50.375, and 60.026 degrees, respectively, matched with *200* -, *220* -, and *311* -planes. The principal peak of *t* -phase was noticed at 2θ of 30.484 degrees, paired with the *101* -plane, while the minor peaks were found at 2θ of 35.597, 50.978, and 60.459 degrees, correspondingly fitted with *110* -, *200* -, and *211* -planes. The chief peak of *m* -phase was identified at 2θ of 28.218 degrees, which equaled *111* -plane, while the petty peaks were found at 2θ of 31.475, 34.196, and 50.167 degrees, singly paired with *(-1)11* , *002* , and *220* planes. The multiplicity of *c* -, *t* -, and *m* -phase varied with zirconia types and sintering rate. The 3Y and 4Y zirconia comprised mainly with *t* -phase (74.4–78.2% and 62.5–70.8%), while 5Y zirconia encompassed principally of *c* -phase (52.8–58.0%). The *m* -phase was the most diminutive phase content in all groups. The sintering rate affected the relative phase contents by increasing the *c* -phase upon raising the sintering rate in the 4Y and 5Y zirconia.

The surface roughness revealed the highest Ra in the 3Y/RS group (5.72 ± 1.16 µm), whereas the lowest Ra in the 5Y/SS group (3.30 ± 0.87 µm). The Ra ranged between 5.72 ± 1.16 and 3.98 ± 0.53 µm for 3Y, between 4.13 ± 0.82 and 4.03 ± 1.09 µm for 4Y, and 3.58 ± 1.09 and 3.30 ± 0.87 µm for 5Y ( Table 2 and Fig. 3D ). The 3D-surface topography of 3Y, 4Y, and 5Y zirconia upon varied sintering rates is shown in Fig. 4J–R . The color gradient represented the level of surface roughness in which the higher areas were demonstrated in red-orange-yellow gradient, whereas the lower areas were demonstrated in green-blue-purple gradient. The Ra tended to decrease from 3Y to 4Y and 5Y zirconia. The sintering rate did not exhibit any influence on Ra.

## Discussion

Achieving esthetic ceramic restoration to reproduce the natural appearance of teeth in clinical practice is based on an inclusive consideration of the essential optical characteristics of the material, including translucency, contrast, opalescence, and color variation. 1 2 This study intended to enhance better optical characteristics of 3Y, 4Y, and 5Y monochromatic zirconia through the sintering procedure by altering the sintering speed. The study showed statistically significant effects of zirconia types and sintering rates as well as their interactions on all optical tested parameters, except ΔE _00_ for the factor of sintering rate, and the interaction of two factors. Hence, the null hypotheses were partly rejected. The study suggested that translucency, contrast, opalescence, and color variation of the monochromatic zirconia were affected by the type of zirconia and sintering speed. This is feasibly related to the complex microstructure of the zirconia as evidenced in other studies. 4 5

Reflecting translucency, the study discovered that the translucency increased as the grain size of zirconia expanded. The quantities of large zirconia grains were perhaps correlated with translucency because they encouraged light transmission and reduced the scattering effect of the incidence light at the grain boundaries as confirmed by other studies. 4 5 This investigation showed that the 5Y containing zirconia presented higher translucency than 4Y and 3Y. This feasibly correlated with the increased amount of Y content that could augment the zirconia grain growth as verified by the SEM photomicrographs and EDS analysis. Similarly, the increasing amount of Y in zirconia may intensify the *c* -phase as indicated by XRD. An isotropic structure of the *c* -phase at the grain boundary possibly improves the light transmission and lessens the light reflection and deflection within the zirconia. 5 11 13 The study also indicated that the less surface roughness, the superior translucency of zirconia presented as evidence of higher Ra of 3Y than 4Y and 5Y. Furthermore, the larger quantity of Y in zirconia and the bigger grain size were demonstrated, which is inversely related to the Ra value. The smooth surface certainly promotes translucence by diminishing the light reflection and deflection effect from the zirconia surface. 10 This study also signified that the variation in the sintering rate affected the translucence of zirconia. The translucency could be promoted by speeding the sintering rate as described in the FS and SS strategy. This is possibly correlated to the grain size enlargement upon FS and SS sintering rate. The rapid rising of temperature through FS and SS protocol possibly steered the simultaneously speedy growth of zirconia grains that feasibly promoted surface integration of the grain boundaries. Accordingly, this occurrence could lead to the pore size declining via the zirconia grains condensation process. 16 26 Therefore, improving the translucency of the zirconia through the FS and SS sintering strategy is feasibly triggered by the grain densification and the pore reduction process that improves the light transmission through the zirconia. The influence of rapid sintering rate on the translucency of zirconia was endorsed by other studies. 3 24 27 Nevertheless, this phenomenon as opposed to other studies, possibly relates to the differences in zirconia brands and sintering protocols. 23 25

The OP values of the restorative materials should be fairly close to the OP values of human enamel to replicate the optical appearance of the natural teeth. Significant differences in OP among types of zirconia and varied sintering rates were denoted in this study. This is perhaps associated with the variation of the pigment substances and the additive chemical compositions that comprise the zirconia blank during the manufacturing process, which cause the difference in light reflection or refraction. The 3Y and 4Y significantly held higher OP than 5Y, which may be associated with the high scattering effect of the *t* -phase that presented as the principal phase component in 3Y and 4Y, compared with 5Y. Likewise, the highest OP was observed in 4Y, likely due to the high amount of iron (Fe) component contained in zirconia. These results align with previous studies, which reported a correlation between OP values and the increasing chroma and value of ceramics. 18 20 Moreover, the study found that the opalescence slightly decreased, but not significantly, while increasing the sintering rate. The finding is probably associated with the microstructure of the zirconia grains that correlated to the effect of light transmittance and reflectance at the grain boundaries. The high transmission of light is associated with low opalescence due to the low light scattering at the grain boundaries and pores between the zirconia grains, as supported by a former study. 28 Even though the OP values observed in this study (2.52–2.81) were lower than the OP values of the human enamel (19.8–27.6), however, they were still within the normal range of the modern dental ceramics (1.6–21.6). 19 28

The color difference value is crucial for determining the amount of color alteration of different types of zirconia upon varying sintering rates. Less ΔE _00_ values indicate less color alteration or better color stability. The study indicated a significant influence of the zirconia types, but not sintering rates on the color difference. Among them, the 3Y had the lowest ΔE _00_ value indicating the least color alteration, while the 5Y had the highest ΔE _00_ value indicating the utmost color alteration. Regarding the sintering rate strategy, the increasing sintering rate seems to initiate color alteration, even though not significant. This might relate to the high translucency appearance of zirconia ceramics that were highly sensitive to color mismatching due to the microstructure of the crystalline phases that could diminish the scattering of light. 18 This existence was proven by previous studies that stated a strong correlation between translucence and color difference of material. 9 Besides, background color and the ceramic thickness also influence ΔE _00_ , especially in the high translucence ceramics. 17 Vice versa, high-opacity zirconia tended to exhibit a true color constantly, due to its masking capability. 17 Nevertheless, all types of zirconia in this study presented the ΔE _00_ values within the acceptability threshold (ΔE _00_ ≤ 1.8), which means clinically acceptable optical appearance. 7 17 The study corresponded with a former study that reported the speed sintering protocol produced the ΔE _00_ lower than the acceptability threshold for most zirconia brands (ΔE _00_ = 1.24–1.59). 11

Based on this present study, the optical characteristics of monochromatic zirconia were influenced by types of zirconia and sintering rates. This inferred that the selection of different sintering rate especially in the speed sintering could be executed for sintering the 3Y, 4Y, and 5Y monochromatic zirconia to improve optical characteristics _._ The rapid sintering through increasing sintering rate can enhance translucency, but slightly reduce opalescence, and tiny color alteration within the acceptability threshold for all types of zirconia. To produce zirconia restoration with enhanced translucency, opalescence, and optimal color alteration, it is recommended to use 5Y monochromatic zirconia, combined with a speeding sintering rate for fabrication restoration in clinical practice. Nevertheless, the study had a distinct limitation since it considered only the influence of the sintering rate on the optical characteristics of 3Y, 4Y, and 5Y monochromatic zirconia. In addition, only one brand of zirconia was selected for the study. The effect of varied sintering rates on the mechanical characteristics, long-term color stability, and the precision of the restoration, fabricated from different brands of zirconia should be further investigated to fulfill the clinical requirement.

## Conclusion

This study indicated that the optical characteristics were influenced by zirconia types and sintering rates. The alteration of sintering rate significantly affected the translucency, contrast, and opalescence of zirconia, with a minute effect on color alteration, depending on the quantities of yttria contained in zirconia. However, adjustment of sintering rates to achieve superior optical characteristics of zirconia with appropriate processing time for chairside restorative treatment was feasible. Sintering monochromatic zirconia with a speedy sintering rate was an intensely effective method than the regular sintering rate to provide an efficient achievement of better translucency, contrast, and opalescence for 5Y than 4Y and 3Y, with clinically acceptable color alteration. Hence, to achieve the most auspicious optical characteristics, the speedy sintering rate was recommended in the sintering process for all types of yttria containing monochromatic zirconia.
